# Combining Donor Characteristics with Immunohistological Data Improves the Prediction of Islet Isolation Success

**DOI:** 10.1155/2016/4214328

**Published:** 2016-10-10

**Authors:** Zuzana Berkova, Frantisek Saudek, Peter Girman, Klara Zacharovova, Jan Kriz, Eva Fabryova, Ivan Leontovyc, Tomas Koblas, Lucie Kosinova, Tomas Neskudla, Ema Vavrova, David Habart, Sarka Loukotova, Martina Zahradnicka, Kvetoslav Lipar, Ludek Voska, Jelena Skibova

**Affiliations:** ^1^Laboratory of Pancreatic Islets, Institute for Clinical and Experimental Medicine, Prague, Czech Republic; ^2^Department of Diabetes, Diabetes Centre, Institute for Clinical and Experimental Medicine, Prague, Czech Republic; ^3^Transplant Surgery Department, Transplant Centre, Institute for Clinical and Experimental Medicine, Prague, Czech Republic; ^4^Department of Clinical and Transplant Pathology, Institute for Clinical and Experimental Medicine, Prague, Czech Republic; ^5^Department of Medical Statistics, Institute for Clinical and Experimental Medicine, Prague, Czech Republic

## Abstract

Variability of pancreatic donors may significantly impact the success of islet isolation. The aim of this study was to evaluate donor factors associated with isolation failure and to investigate whether immunohistology could contribute to organ selection. Donor characteristics were evaluated for both successful (*n* = 61) and failed (*n* = 98) islet isolations. Samples of donor pancreatic tissue (*n* = 78) were taken for immunohistochemical examination. Islet isolations with 250000 islet equivalents were considered successful. We confirmed that BMI of less than 25 kg/m^2^ (*P* < 0.001), cold ischemia time more than 8 hours (*P* < 0.01), hospitalization longer than 96 hours (*P* < 0.05), higher catecholamine doses (*P* < 0.05), and edematous pancreases (*P* < 0.01) all unfavorably affected isolation outcome. Subsequent immunohistochemical examination of donor pancreases confirmed significant differences in insulin-positive areas (*P* < 0.001). ROC analyses then established that the insulin-positive area in the pancreas could be used to predict the likely success of islet isolation (*P* < 0.001). At the optimal cutoff point (>1.02%), sensitivity and specificity were 89% and 76%, respectively. To conclude, while the insulin-positive area, determined preislet isolation, as a single variable, is sufficient to predict isolation outcome and helps to improve the success of this procedure, its combination with the established donor scoring system might further improve organ selection.

## 1. Introduction

Type 1 diabetic patients require intensive insulin therapy to avoid serious complications caused by high blood glucose levels. A small number of diabetic patients experience a very labile form of diabetes with unpredictable and repeated hypoglycemia. The transplantation of pancreatic islets to these patients positively influences metabolic control and helps to prevent hypoglycemic episodes. Insulin independence in diabetic patients can be achieved by whole organ transplantation or by the transplantation of isolated islets from 2 or 3 donors [1], although single-donor infusion can also reverse the requirement for exogenous insulin [2].

Despite continuing progress in the practice of pancreatic islet isolation, isolation outcome is highly variable and remains hard to predict. Currently, appropriate donor selection is seen as one of the keys to successful pancreatic islet isolation. The Edmonton group developed a scoring system [3] to predict the suitability of potential pancreatic donors prior to organ processing. The ability to accurately reject poor quality donor pancreases enhances overall isolation success and decreases the costs that result from failed isolations [3, 4]. Recently, a second scoring system (North American Islet Donor Score) which includes some different variables to predict isolation success has also been described.

Because of a shortage of organ donors in the last few years, which probably reflects a greater number of people on the waiting list, there are renewed efforts to improve islet isolation from valuable single-donor pancreases. According to previously published studies, the variability of pancreatic donors may significantly influence the success with which we can subsequently isolate islets. The following donor characteristics have all been identified as being critical for isolation outcome: age [3, 5–8], body mass index [5, 6, 8, 9], cardiac arrest including hypotensive episodes and the dosage of vasopressors [5, 9, 10], blood glucose [5, 10], and amylase levels [9]. Moreover, the procurement team [5, 7–10], duration of enzymatic digestion, or type of enzyme used [7, 8, 10], together with the duration of cold ischemia [5, 6, 9], are also important criteria in contributing to the variability of isolation outcome.

Currently, in most centers, pancreases that are unsuitable for solid organ transplantation (in terms of age, BMI, or other factors) are allocated for islet isolation. The decision as to whether to accept or decline an organ for islet isolation is based on donor characteristics together with a later macroscopic evaluation of the donor pancreas. Unfortunately, islet yield, which is the commonly used indicator of a successful isolation, is largely dependent on the islet mass in the pancreas prior to islet isolation and cannot be accurately determined ahead of pancreas processing.

Precise and effective donor selection is needed to avoid discarding potentially useful donations or conversely accepting those with inadequate islet mass. The purpose of this study was to evaluate the donor and pancreas characteristics that influence the outcome of islet isolation and to investigate the value of immunohistological examination of donor pancreases for organ selection.

## 2. Materials and Methods

### 2.1. Human Islet Isolation

Human pancreases were recovered from 173 deceased donors after brain-dead from January 2010 until December 2014. Accepted but not processed pancreases were excluded (*n* = 14). Pancreatic islets were isolated from 159 donors according to a modified semiautomatic Ricordi's method [11]. All protocols were approved by the Ethics Committee of the Institute for Clinical and Experimental Medicine and Thomayer's Teaching Hospital. Pancreatic islet isolation (*n* = 8) for autologous transplantation was also an exclusion criterion.

Flushed organs were removed with the spleen and a portion of the duodenum and transported on ice to the islet isolation laboratory. The pancreas was processed on a homemade cooling pad. The spleen, duodenum, and adipose tissue were removed, the main pancreatic duct was cannulated, and the organ was perfused with collagenase solution; Collagenase NB1 (Serva Electrophoresis GmbH, Germany), or CIzyme™ collagenase HA (VitaCyte, USA) were used. The distended organ was then placed into a digestion chamber at a controlled temperature, with enzyme solution recirculation. During the digestion phase, small samples of the digested tissue from the chamber were collected, stained with diphenyl-thiocarbazone (Sigma-Aldrich, USA), and microscopically evaluated in real time. Digestion was stopped by cooling and dilution, with the digested pancreatic tissue collected in the washing solution. After pooling, the tissue was placed on ice in University of Wisconsin (UW) solution for approximately 1 hour. A discontinuous gradient was used to determine the pancreatic tissue density and the appropriate density range for separation. Pancreatic islets were separated from exocrine tissue by centrifugation in a continuous Biocoll gradient (Biochrom GmbH, Germany), using a COBE® 2991 Cell Processor (Terumo BCT, USA). Islet containing fractions were collected, washed, and counted. Isolated islets were then cultured in supplemented CMRL-1066 medium (PAN-Biotech GmbH, Germany), in a humidified incubator at 37°C and a 5% CO_2_ atmosphere.

The minimum islet mass considered for islet transplantation is 4000 islet equivalents (IE) per kg body weight of the recipient. Therefore pancreatic islet isolations with islet yields of ≥250 000 IE, a purity of at least 30%, and viability greater than 80%, were considered successful. The quality of isolated islets was assessed by live/dead cells differential staining and by glucose stimulated insulin secretion, as previously described [12]. Briefly, islet vitality was based on the cell membrane integrity test after staining with propidium iodide (dead red cells) and acridine orange (live green cells). The in vitro function of the isolated islets was measured as insulin release in Kreb's solution with low glucose (3 mmol/L), high glucose (22 mmol/L), and then low glucose again. At the end of each incubation period, an aliquot of medium was collected for insulin radioimmunoassay using ^125^I RIA Kit (ICN Pharmaceuticals, USA). Results were expressed as stimulation indices.

### 2.2. Assessment of Donor Points

Based on the Edmonton scoring system the selected donor characteristics of age, BMI, cold ischemia time (CIT), the cause of death, intensive care hospitalization, the levels of serum amylase, use of vasopressors, blood glucose levels, organ procurement, and social and medical history were evaluated. The maximum score is 100 donor points (DP), while the selected organ characteristics of size, consistency (edema or fibrosis), damage, quality of flush, procurement, and packaging only serve to negatively influence the donor score. Based on the final score, donors were divided into 6 categories: poor donors (0–49.5 DP), marginal donors (50–59.5), intermediate donors (60–69.5 and 70–79.5 DP), and optimal donors (80–89.5 and 90–100 DP) [3]. The frequency of donors in each category together with the frequency of successful isolations was collated.

### 2.3. Histological and Immunohistochemical Examination

From August 2011, pancreatic tissue samples were recovered for histological examination. Pancreatic tissue was taken from the head or neck of the donor pancreas depending on whether one or two cannulas for collagenase perfusion had been applied. The samples were fixed in 10% formaldehyde with chalk at 4°C overnight. Routine hematoxylin & eosin staining was performed for basic histological assessment. For insulin detection, immunohistochemical staining was used. Four *μ*m thick sections were deparaffinized in xylene and rehydrated in a graded ethanol series. After rinsing in 0.2% Triton X 100 and PBS, endogenous biotin was blocked using the biotin blocking system (DakoCytomation, Denmark), with endogenous peroxidase blocked with 0.3% H_2_O_2_ in 70% ethanol. To prevent nonspecific binding, samples were preincubated with 10% horse serum. For beta cell detection, specimens were incubated with a primary anti-insulin antibody (Sigma-Aldrich, USA) and detected using a Histofine Simple Stain Rat MAX PO (NICHIREI, Japan). After incubation with Dako Liquid DAB+ Substrate-Chromogen System (DakoCytomation, Denmark), specimens were counterstained with hematoxylin. Pancreatic tissue slices (*n* = 78) were scanned using the EVOS FL Auto imaging system (Thermo Fisher Scientific Inc., USA). Images of entire sections were analysed using the ImageJ software (Wayne Rasband, NIH, USA). Pancreatic tissue was manually outlined and insulin-positive cells were selected using the color threshold tool. The percentage of insulin-positive area to total pancreatic area was then calculated.

### 2.4. Statistical Analyses

Results are expressed as mean ± SD. To test for a normal distribution of our data, the Lilliefors test for normality was performed. Differences between groups were compared using the Mann–Whitney *U* test or Student's *t* test; differences between proportions were compared using the Chi-square test. To predict isolation outcome based on immunohistochemical evaluation and donor score, receiver operating characteristic (ROC) analyses were performed and area under the curve (AUC) values were calculated. The associations between selected donor characteristics including the area of insulin staining and donor score were analysed using the Pearson correlation coefficient. Multivariate stepwise logistic regression analysis was then used to determine isolation success based on these two criteria. A *P* value <0.05 was considered to indicate statistical significance.

## 3. Results

From January 2010 until December 2013 our isolation center received 147 donor pancreases for islet isolation. Because of damage during procurement, 8 pancreases were rejected. 139 donor pancreases were isolated and retrospectively evaluated to assess donor points (see Table 1). The frequency of poor donors (defined as DP of 0 to 49.5) was 10.1%, with 21.4% of isolations from these donations being successful. A donor frequency of 17.3% was seen for marginal donor pancreases (DP 50 to 59.5), with a 41.7% success rate. The highest donor frequencies of 33.8% and 27.3% were achieved for organs with DP ratings of 60 to 69.5 and 70 to 79.5, for which the frequencies of isolation success were 34.0% and 36.8%, respectively. While the frequency of optimal donors (DP 80 to 89.5) was only 11.5%, the success of islet isolation for these donations was highest, at 56.3%. The overall success rate from 2010 to 2013 was 37.4%. A comparison of DP showed no significant differences 67.6 ± 11.3 DP for the successful group versus 64.6 ± 11.8 DP for the failed group (*P* > 0.05).

In 2014, 26 donors were accepted for pancreatic islet isolation. According to the results from our retrospective study, exclusion of poor donors at our center was prohibited given that just over a fifth (21.4%) of islet isolations from these donors were successful. Donor selection was therefore based primarily on previous experience, which led to the rejection of 5 edematous pancreases. Moreover, one pancreas was rejected because of a procurement-related issue. We found that islet isolation from poor or marginal donor pancreases (donor frequencies of 15% and 10%, resp.) was unsuccessful. The frequency of donors with a DP rating of 60 to 69.5 was 15%; from these, 66.7% of islet isolations were successful. The most frequent donor group (45%) was the 70 to 79.5 DP group, for which successful isolations were achieved in 44.4% of cases. Islet isolations from optimal donors (15%) were all successful. In 2014, our overall success rate increased to 45.0%. Over the last 5 years, any donor with a DP of more than 90 was available for pancreatic islet isolation in our center.

The number of islet isolations per year has continuously decreased in our isolation center (from 46 in 2010 to 20 in 2014); in average we had 32 isolations annually. The average islet yield from all 159 isolations was 223 289 ± 131 276 IE, while 61 successful isolations produced 356 328 ± 87 356 IE. The mean purity of the transplanted islets was 49.7 ± 8.0%. Islet transplantation was performed for 50 recipients; 19 patients received a single infusion, 11 patients received two infusions, and 3 patients received three islet infusions. In vitro analyses of the isolated islets showed comparable viability of the transplanted and nontransplanted islets at 94.9 ± 4.2% versus 91.0 ± 7.1%, respectively (*P* > 0.05). However, the quality of the islets, as assessed by glucose stimulated insulin release, revealed significant differences. The stimulation indexes of the transplanted versus nontransplanted islets were 7.6 ± 5.5 versus 5.8 ± 6.0, respectively (*P* < 0.01).

To identify the critical factors associated with isolation outcome, selected donor characteristics were retrospectively analysed in successful (*n* = 61) and failed (*n* = 98) islet isolations (see Table 2). While no differences were observed in terms of gender, age, cause of death, blood glucose levels, amylase level, and type of collagenase used, significant differences were found for body mass index, body surface area (BSA), duration of cold ischemia, length of intensive care hospitalization, and usage of vasoactive drugs. A BMI of less than 25 kg/m^2^ was strongly associated with islet isolation failure (*P* < 0.001), while islet isolations from donors with a BMI less than 20 kg/m^2^ (*n* = 8) were completely unsuccessful (*P* < 0.05). Higher doses of vasoactive drugs (*P* < 0.05), hospitalization in excess of 96 hours (*P* < 0.05), and cold ischemia for more than 8 hours (*P* < 0.01) all had adverse effects on islet isolation. Selected pancreatic characteristics such as weight, fatty infiltration, and capsule injury were similar for both groups (*P* > 0.05), although significant differences were observed in pancreas consistency; edematous pancreases negatively influenced isolation outcome (*P* < 0.01). Digestion time was also strongly associated with the isolation outcome (*P* < 0.01).

From August 2011 tissue samples of donor pancreases were recovered and processed for histological and immunohistochemical examination. Damaged samples were excluded from the study (*n* = 3). Histological examination of donor pancreases was performed on 78 samples obtained prior to islet isolation. Basic histological examination of donor pancreases showed no direct impact on isolation outcome (see Table 3). Although edema can negatively influence isolation outcome, this difference was not statistically significant.

**Figure 1:**
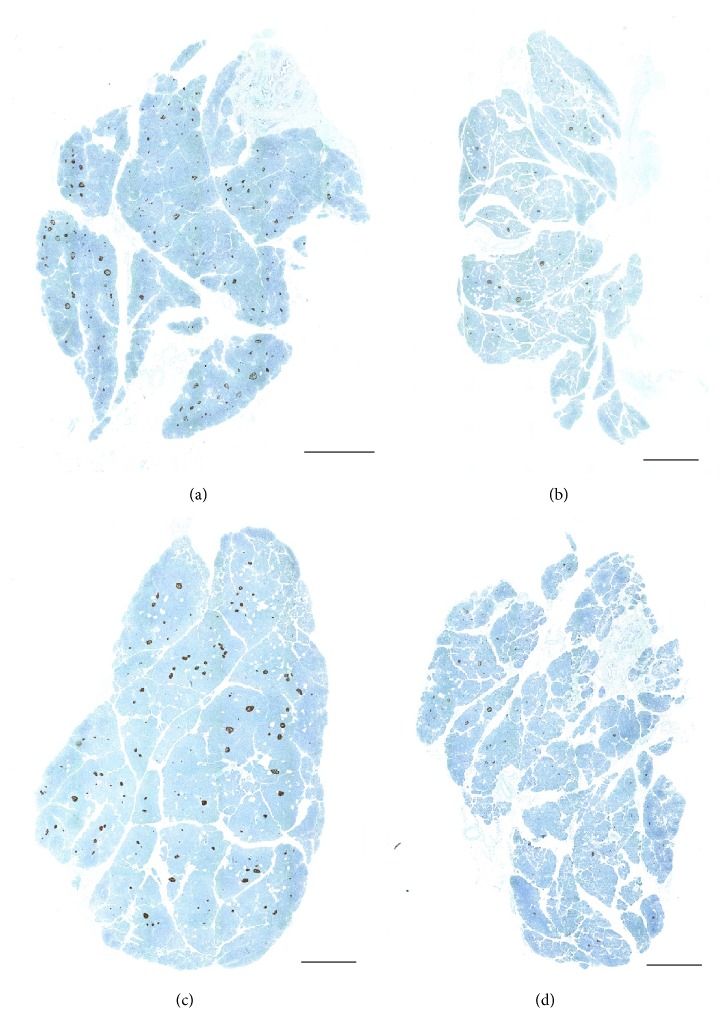
Representative immunohistochemical stains of donor pancreases from successful (a, c) and failed isolations (b, d). The percentage of insulin-positive area was (a) 1.9%, (b) 0.74%, (c) 1.5%, and (d) 0.64%. The scale bar denotes 2 mm.

**Figure 2:**
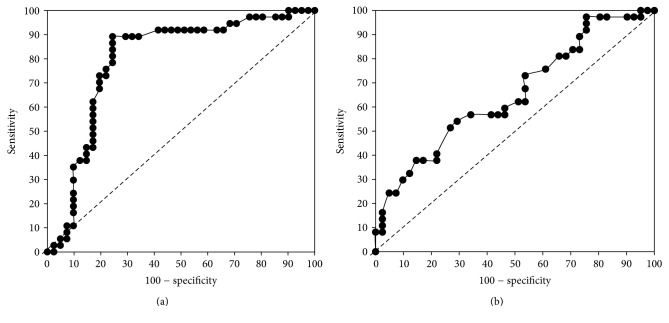
Receiver operating characteristic (ROC) curve for predicting islet isolation success. (a) Area under the curve (AUC) = 0.796 for immunohistochemistry data; 95% confidence interval, 0.689 to 0.879; *P* < 0.001. Using an optimal cutoff point for an insulin-positive area in the pancreas of >1.02% resulted in 89% sensitivity and 76% specificity. (b) Area under the curve (AUC) = 0.653 for donor score; 95% confidence interval, 0.537 to 0.758; *P* < 0.05. The optimal cutoff point using a donor score of >68 points resulted in 60% sensitivity and 54% specificity. Prediction of islet isolation outcome was therefore superior using the immunohistochemical data (*P* = 0.011).

Analyses of immunohistochemical data showed that successful isolations were achieved from organs with an increased insulin-positive area. Representative micrographs of pancreatic tissue are shown in Figure 1. The percentage of the insulin-positive area in the successful group (*n* = 37) was 1.43 ± 0.6, significantly higher than that for the failed group (*n* = 41), which was 1.02 ± 0.7 (*P* < 0.001). An increased insulin-positive area was therefore strongly associated with a successful isolation outcome. To demonstrate the ability to predict isolation outcome using this criterion, a ROC analysis was performed using the insulin-positive areas determined for donor pancreases (see Figure 2(a)). This analysis confirmed a statistically significant relationship: the area under the ROC curve (AUC) = 0.796; 95% confidence interval: 0.689 to 0.879; *P* < 0.001. An optimal cutoff point for the insulin-positive area in the pancreas of >1.02% generated accuracy-related values of 89% for sensitivity and 76% for specificity. The predictability of isolation outcome based on donor score also showed statistical significance, although to a lesser extent: AUC = 0.653; 95% confidence interval: 0.537 to 0.758; *P* < 0.05 (see Figure 2(b)). The optimal cutoff point for a donor score of >68 points corresponded to values of 60% for sensitivity and 54% for specificity. Comparison of the AUCs from both analyses showed the significantly greater predictive power of immunohistochemical examination (*P* < 0.05).

**Figure 3:**
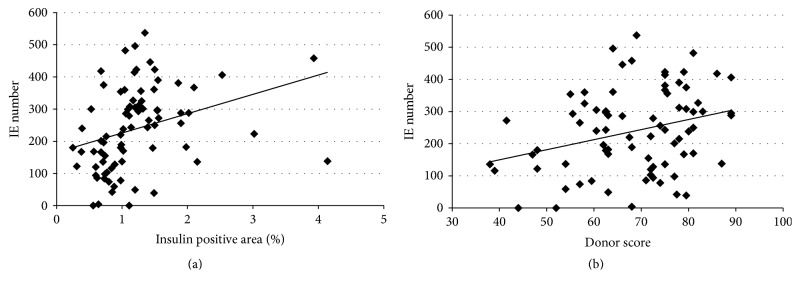
The number of isolated islet equivalents positively correlated with (a) the insulin-positive area in the pancreas (*r* = 0.318; *P* < 0.01) and (b) donor score (*r* = 0.298; *P* < 0.01).

Selected donor characteristics including immunohistological data and donor score were then analysed using the Pearson* r* correlation coefficient (see Figure 3). The results showed that IE number positively correlated with the immunohistological data (*r* = 0.318, *P* < 0.01), BMI (*r* = 0.255, *P* < 0.05), BSA (*r* = 0.364, *P* < 0.001), and donor score (*r* = 0.298, *P* < 0.01) and negatively with digestion time (*r* = −0.321, *P* < 0.01). Multivariate stepwise logistic regression was then performed to show the predictability of isolation outcome with the multivariable use of the immunohistochemistry data with the donor scoring system (see Table 4). This model with an optimal *P* value cutoff point >0.310 had sensitivity 97% and specificity of 34%.

## 4. Discussion

The clinical islet program at our isolation center was initiated in the late nineties with our first islet transplantation carried out in 2005. Despite considerable progress since then, isolation results still remain inconsistent and unpredictable. “Optimal” donor pancreases are allocated for solid organ transplantation, with only those deemed unsuitable for transplantation processed for islet isolation. Therefore more effective donor selection could be a promising tool to increase our success rate for islet isolation.

Pancreatic islets are separated from the surrounding pancreatic tissue by a capsule of connective tissue fibers, which is contiguous with the exocrine tissue. Collagen distribution within the pancreatic parenchyma is important for collagenase digestion and postpurification islet recovery. The currently used indicator of successful islet isolation is islet yield, which is an unknown quantity before the procedure. However, given that the number of islets in the pancreas can considerably influence isolation outcome, a prior evaluation of pancreas histology, to reveal the number and shape of islets, could be helpful in reaching a decision as to whether to process the pancreas or not.

To the best of our knowledge, only two studies have investigated the histology of donor pancreases in the previous two decades. The first of these, published by Mahler et al. [13], analysed the morphologic and histopathologic characteristics of 109 donor pancreases. It was determined that well demarcated islets (from the surrounding exocrine tissue), as well as fat content, were crucial criteria in predicting successful islet isolation. In the second study, published by Hanley et al. [7], 41 randomly selected pancreas samples were evaluated and a positive correlation was found between beta cell volume and isolation yield. The results of our study confirm that successful islet isolation is achieved from organs with larger insulin-positive areas. Although the predictive value of immunohistology is quite high [14], it is still insufficient insofar as it may result in the accidental rejection of suitable donors. Multivariate stepwise logistic regression analyses showed that, by combining immunohistology with the already established donor scoring system, we could significantly increase the accuracy of our predictions. According to our results, screening could eliminate a third of donations destined to be unsuccessful for islet isolation, at the cost of one successful pancreas.

Overnight formaldehyde fixation of pancreatic samples precludes any histological evaluation before pancreas processing because of the prolonged cold ischemia time. Instead, this long-term fixation step could be substituted by cryopreservation. The use of frozen samples can reduce the overall processing time from almost 2-3 days to only 2 hours for immunohistochemistry or 30 minutes for dithizone staining.

According to previously published studies, many islet isolation centers evaluate donor variables to identify predictors of islet isolation outcome [3–7, 9, 10] and to a lesser extent variables that may influence transplantation outcome [8, 15]. By examining donor- and pancreas-related characteristics we found that the most influential factors in the success of islet isolation were BMI, BSA, cold ischemia time, vasoactive drug dosage, hospitalization time, and pancreas consistency.

The most commonly described variable to influence the outcome of islet isolation is donor age [5–8], although numerous discrepancies for this metric exist in the literature, possibly reflecting the varying age ranges for younger and older donors. Reduced islet yield was observed from donors of less than 20 years of age [5, 6], while a higher donor age was associated with higher islet yields. In our isolation center, 65% of successful isolations were achieved from donors aged 45–50 years, falling to 31% for donors aged less than 45, dropping further still, to 20%, for donors under 20 years of age. These results could be explained by age related differences in collagen composition and distribution in the pancreas and impaired collagenase efficiency for younger pancreases. Despite the increased success of islet isolation from older donors, their islet function was diminished to the extent that the optimal donor age in terms of transplantation outcome is 20–45 years of age, as published by Niclauss et al. [15].

Positive correlations between BMI and isolation success have been consistently reported [5, 6, 8, 16]. Our results confirm this observation; islet isolations from donors with a BMI higher than 25 kg/m^2^ were successful in almost 53% of cases, while the success rate from donors with a BMI < 25 kg/m^2^ fell to only 21%. Interestingly, in the Edmonton scoring system, the most valuable donors have a BMI of 25–30 kg/m^2^, whilst the North American Islet Donor Score (NAIDS) provides positive evaluations for BMI values up to 52 kg/m^2^. We can expect that donors with higher BMI have larger pancreases that contain more islets. Although pancreatic weight cannot be assessed before pancreas acceptance, the study of Kin et al. [17] showed that pancreas weight correlated well with donor weight and body surface area. In comparison to the Edmonton scoring system, the NAID score evaluates BSA and donor weight as important donor variables that can improve donor selection.

Donor organ preservation has been the subject of multiple studies although the results are controversial. Qin et al. [18], in multicenter analyses, compared the University of Wisconsin (UW) solution and the two-layer method (TLM) for pancreas storage and concluded that the TLM was beneficial for prolonged pancreas preservation, while short-term preservation was comparable with either method. Our isolation center for organ procurement uses a Histidine-Tryptophan-Ketoglutarate (HTK) solution. The reduced survival of grafts derived from pancreases preserved in HTK has been reported by Stewart et al. [19], while a recent study of Paushter et al. [20] demonstrated equivalent effectiveness with UW versus HTK preservation. Islet recovery can be significantly influenced by cold ischemia time. In our study, a CIT of longer than 8 hours was strongly associated with isolation failure. This observation was in agreement with previously published data for long-term pancreas storage in UW solution [5, 6] and for HTK preservation prior to isolation [21].

After organ acceptance, donor pancreases are macroscopically evaluated in an islet isolation laboratory. Based on previously published data, a large pancreas probably associated with a higher BMI, as well as fatty infiltration, increases the success of islet isolation [7]. A higher fat content also correlated positively with islet yield in the study published by Mahler et al. [13]. However in our study, no differences for pancreas weight and fatty infiltration were observed. Additionally, damage to the pancreatic capsule was not associated with isolation failure, as reported by Sakuma et al. [22]. According to our results, the most important pancreatic characteristic to influence islet isolation was edema. In edematous pancreases, collagenase perfusion could be impaired with prolonged digestion times diminishing isolation success. Therefore the exclusion of edematous pancreases in our center considerably increased isolation success ratios.

## 5. Conclusions

In conclusion, the size of insulin-positive areas in donor pancreases is strongly associated with the islet isolation outcome. Therefore immunohistological examination of donor pancreases might be helpful in guiding organ selection. We found that the prediction of isolation outcome could be improved by combining immunohistological examination with the donor scoring system. A caveat to this is that while this method cannot eliminate all unsuccessful donors, it can enhance isolation success and at the same time decrease costs resulting from failed isolations.

## Figures and Tables

**Table 1:** Pancreatic donors and successful isolations from 2010 to 2013.

Donor points	Number of donors	Frequency of donors (%)	Number of successful isolations	Frequency of successful isolations (%)
0–49.5	14	10.1	3	21.4
50–59.5	24	17.3	10	41.7
60–69.5	47	33.8	16	34.0
70–79.5	38	27.3	14	36.8
80–89.5	16	11.5	9	56.3
90–100	0	0.0	—	—

Total	139		52	37.4

**Table 2:** Comparison of donor characteristics for successful and failed islet isolations (^*∗*^ *P* < 0.05, ^*∗∗*^ *P* < 0.01, and ^*∗∗∗*^ *P* < 0.001).

	Successful isolations (*n* = 61)	Failed isolations (*n* = 98)	*P* value
Gender (F/M)	17/44	38/60	0.16
Age (years)	47.7 ± 11.3	45.3 ± 13.1	0.36
BMI (kg/m^2^)	27.5 ± 4.1	25.4 ± 4.4	0.0003^*∗∗∗*^
BMI (<25 kg/m^2^)	15/61	56/98	0.00006^*∗∗∗*^
BSA (m^2^)	2.06 ± 0.2	1.93 ± 0.2	0.0004^*∗∗∗*^
Cause of death (traumatic/nontraumatic)	17/44	21/77	0.36
Cold ischemia time (>8 hours)	6/61	29/98	0.004^*∗∗*^
Intensive care hospitalization (days)	3.5 ± 2.7	4.5 ± 3.0	0.009^*∗∗*^
Hospitalization (>96 hours)	16/61	45/98	0.01^*∗*^
Vasoactive drugs (>20 units)	12/61	36/98	0.02^*∗*^
Amylase levels (>2x normal levels)	3/47	10/76	0.24
Blood glucose level (mmol/L)	7.7 ± 2.0	7.9 ± 2.0	0.56
Pancreas weight (g)	143.8 ± 45.7	135.2 ± 42.5	0.32
Pancreas consistency (edema)	0/61	10/98	0.01^*∗∗*^
Collagenase (Serva/VitaCyte)	44/17	74/23	0.56
Digestion time (min)	19.1 ± 6.7	24.1 ± 9.9	0.0013^*∗∗*^
Digestion (>20 min)	25/61	62/98	0.006^*∗∗*^

**Table 3:** Histological comparison of donor pancreases in successful and failed islet isolations.

	Successful isolations (*n* = 37)	Failed isolations (*n* = 41)	*P* value
Fat content	17/37	16/41	0.54
Fibrosis	15/37	17/41	0.93
Edema	0/37	4/41	0.05
Necrosis	3/37	6/41	0.37
Inflammation	4/37	6/41	0.61

**Table 4:** Stepwise multivariate logistic regression of factors predicting isolation success.

	Coefficient	Odds ratio	95% CI
Immunohistology	1.107	3.03	1.11–8.28
Donor score	0.041	1.04	0.998–1.09
Constant	−4.2		
